# A Practical Treatise on the Venereal Disease

**Published:** 1841-04-01

**Authors:** 


### A Practical Treatise on the Venereal Disease. Founded on Six Lectures on that Subject, delivered in the Session of 1838-39, at the Aldersgate School of Medicine. With Plates. By *F. C. Skey*, F.R.S. &c. 12mo. pp. 195. Three Plates. London, 1841.

Mr. Skey appears to be a staunch advocate for the self-generation of venereal diseases. He says,—

"The term self-generated (I will not say spontaneous, for that is still more objectionable) expresses something short of the idea I wish to convey. I mean that, in a certain condition of constitution, the elements of a poison lie dormant, which may be developed by the action of a simple irritant, and that that irritant may exist in the form of any apparently simple, but unhealthy exciting cause in the female, such as leucorrhœa, menstrual fluid, or indeed any impure secretion of a puriform character; it may also be developed by mechanical irritation." 4.

Certainly Mr. Skey deserves a testimonial "by general subscription of the ladies;" for his doctrine, carried out, would be to them charity, covering a multitude of sins.

"If we adopt the practice of inquiry into all the cases which come under our eye, occurring in persons in a respectable station of life, and of course worthy of credit, it is remarkable how frequently patients themselves express their astonishment at becoming the subjects of disease. And well may they express astonishment, and marvel at its existence in their own persons, for there is often, I am persuaded, no other ground for the supposition of disease in the female, who is supposed to have produced it, than is sanctioned by prejudice, and by a too implicit confidence in the doctrines of our progenitors." 5.

It appears to us that Mr. Skey carries his notion of the self-generating properties of venereal diseases à l'outrance. Did they exist to the extent he argues for, we really conceive that not a married couple in Great Britain would be safe. Yet, amongst those married persons, it does so happen, that venereal disease seldom occurs without there being a pretty good reason for it.

Mr. Skey professes that he is "a thorough believer in the plurality of poisons." It is difficult to understand the construction of the following passage, but the author's meaning is obvious.

"I can distinguish, to my entire satisfaction, at least three distinct forms of sore, succeeded by three as distinct results; and I should stare with wonder, and with increased admiration, at the infinite variety of nature's products, were I to discover in any particular case a direct departure from the general laws which appear to me, to govern them. But these laws exist but for a period. The distinction of diseases which appertain to this subject at the present day, in virtue, and that onward march from maturity to decay, from which disease itself is scarcely exempt, will probably be inapplicable at a future one. I do not so much dwell on the liabilities of individuals to a peculiar character of sore, although I consider this subject by no means unimportant, as I attach importance to the doctrine I shall afterwards endeavour to inculcate, that each sore has peculiar results, incidental to neglect of treatment." 19.

The three kinds of venereal sores admitted by Mr. Skey are—1, The common sore, or venerola vulgaris of Mr. Evans; 2, the phagadænic sore; and, 3, the indurated sore of Mr. Hunter. These and their modifications include all forms of venereal ulcers.

Mr. Skey thus speaks of mercury in the venerola vulgaris—

"As a general rule, there is no necessity for the administration of mercury, in any form or quantity. At the same time you need not forswear its use. In moderate quantities, it is inoffensive and unobjectionable, and may often contribute to the healthy progress of the sore. Five grains of blue pill to an ordinary patient, not the subject of mercurial idiosyncracy, may accelerate the cure, when given each, or alternate nights; but it should not be used continuously for more than a few days. There is no advantage in what is called 'touching the gums;' but generally a great disadvantage both to the sore, and to the health." 47.

With an extract containing Mr. Skey's treatment of the sloughing sore—treatment which we confess, we are not ourselves partial to—we conclude. There is much in the work which may admit of question, but as it has been already published in another journal, we cannot devote further space to it in this. We recommend it to our readers.

"The treatment is essentially antiphlogistic, and that, almost without reference to its probable injury to the future health of the individual. The destructive process is so rapid, and the value of the organ so great, that no expense can be deemed exorbitant, with which to purchase even temporary relief. If the disease be external, and accessible to local means, even the antiphlogistic treatment must yield to more direct means of arresting the destructive actions of the sore. This may be effected by the free and unsparing application of the undiluted nitric acid, which must be carefully but extensively applied on every part of the gangrenous surface, till the whole is converted into a soft white crust. This may be followed by a full dose of laudanum, and the crisis of the disease is accomplished. When the sloughing action is confined within the phymosed prepuce, and of course inaccessible to local means, as large a quantity of blood must be taken by venæsection as the patient will bear, accompanied by the exhibition of full doses of mercury both internally and by inunction, with the view to affect the system as early as possible—not with the intention to kill a poison, but to arrest inflammation, of which the gangrene is the immediate product. Frequent ablution, by injection of warm water, or strong decoction of poppy heads, by means of a syringe, should be employed for the purpose of dislodging any disengaged portion of the gangrenous mass that may be separating; and cold water, or a bread-and-water poultice, as may best suit the fancy or the reasoning of practitioner, should be applied around the penis." 61.

